# Establishing Polycistronic Expression in the Model Microorganism *Ustilago maydis*

**DOI:** 10.3389/fmicb.2020.01384

**Published:** 2020-06-24

**Authors:** Kira Müntjes, Magnus Philipp, Lisa Hüsemann, Nicole Heucken, Stefanie Weidtkamp-Peters, Kerstin Schipper, Matias D. Zurbriggen, Michael Feldbrügge

**Affiliations:** ^1^Institute for Microbiology, Cluster of Excellence on Plant Sciences, Bioeconomy Science Centre, Heinrich Heine University Düsseldorf, Düsseldorf, Germany; ^2^Institute of Synthetic Biology, Cluster of Excellence on Plant Sciences, Bioeconomy Science Centre, Heinrich Heine University Düsseldorf, Düsseldorf, Germany; ^3^Centre for Advanced Imaging, Heinrich Heine University Düsseldorf, Düsseldorf, Germany

**Keywords:** 2A peptide, FRET, mannosylerythritol lipid, RNA transport, RRM, *Ustilago maydis*

## Abstract

Eukaryotic microorganisms use monocistronic mRNAs to encode proteins. For synthetic biological approaches like metabolic engineering, precise co-expression of several proteins in space and time is advantageous. A straightforward approach is the application of viral 2A peptides to design synthetic polycistronic mRNAs in eukaryotes. During translation of these peptides the ribosome stalls, the peptide chain is released and the ribosome resumes translation. Thus, two independent polypeptide chains can be encoded from a single mRNA when a 2A peptide sequence is placed inbetween the two open reading frames. Here, we establish such a system in the well-studied model microorganism *Ustilago maydis*. Using two fluorescence reporter proteins, we compared the activity of five viral 2A peptides. Their activity was evaluated *in vivo* using fluorescence microscopy and validated using fluorescence resonance energy transfer (FRET). Activity ranged from 20 to 100% and the best performing 2A peptide was P2A from porcine teschovirus-1. As proof of principle, we followed regulated gene expression efficiently over time and synthesised a tri-cistronic mRNA encoding biosynthetic enzymes to produce mannosylerythritol lipids (MELs). In essence, we evaluated 2A peptides *in vivo* and demonstrated the applicability of 2A peptide technology for *U. maydis* in basic and applied science.

## Introduction

In bacteria, gene expression is structured in operons containing polycistronic mRNAs encoding multiple proteins. This has the clear advantage that expression of several proteins can be regulated synchronously using a single promoter and terminator. In eukaryotes, mRNAs are mostly monocistronic and therefore synthesis of each protein can be fine-tuned in space and time. According to the RNA operon model, expression of eukaryotic mRNAs is co-regulated by RNA-binding proteins that determine when and where the corresponding target mRNAs are translated (Keene, 2007). However, for genetic and metabolic engineering it is advantageous to mimic polycistronic mRNAs in eukaryotes for efficient co-regulation of mRNAs in a defined spatio-temporal manner. This circumvents, for example, the multiple uses of identical promoters and terminators, which might reduce overall promoter activity or could interfere with strain generation using homologous recombination (de Felipe et al., 2006; Unkles et al., 2014).

A straightforward approach is the use of viral 2A peptides (de Felipe et al., 2006). These short peptide motifs were first discovered in the foot-and-mouth disease virus (FMDV, F2A peptide) of the *Picornaviridae* virus family (Ryan et al., 1991). Translation of polypeptides containing 2A motifs results in the separation of long viral open reading frames in two units without disassembly of the ribosome (Atkins et al., 2007; Sharma et al., 2012). In a so-called “stop and carry on” mechanism, eukaryotic ribosomes pause at a defined glycine of the characteristic DXEXNPG P motif. The 2A sequence most likely adopts an unfavourable conformation in the exit tunnel, which impairs peptide bond formation between glycine at the P site and the weak nucleophilic amino acid proline at the A site. To overcome ribosomal stalling the translated upstream polypeptide chain with the 2A peptide at its C-terminus is released and translation of the downstream open reading frame carries on using proline as its starting point (Ryan et al., 1991; Atkins et al., 2007).

This ribosomal mechanism does not function in prokaryotes (Donnelly et al., 1997). However, it is widely applicable in eukaryotes as the activity of 2A peptides has been demonstrated in several organisms ranging from plants to animals and fungi (Halpin et al., 1999; Provost et al., 2007; Kim et al., 2011; Daniels et al., 2014; Unkles et al., 2014; Geier et al., 2015). This allows a broad application of 2A peptides to establish polycistronic gene expression in applied science, for example, in the production of carotenoids in plants (Ha et al., 2010), monoclonal antibodies in animal cell culture (Chng et al., 2015) or natural products in fungi (Ryan et al., 1991; Sharma et al., 2012; Beekwilder et al., 2014; Unkles et al., 2014; Souza-Moreira et al., 2018). The latter includes the production of (i) carotenoids in *S. cerevisiae* (Beekwilder et al., 2014), (ii) β-lactam antibiotics, (iii) psychotropic mushroom alkaloids or (iv) a germination inhibitor in *Aspergillus nidulans* (Unkles et al., 2014; Hoefgen et al., 2018; Stroe et al., 2020) as well as (v) fungal toxins in *A. niger* (Schuetze and Meyer, 2017).

We are studying *Ustilago maydis*, the causative agent of corn smut disease (Kahmann and Kämper, 2004; Brefort et al., 2009). Essential for pathogenicity is a morphological switch from yeast to hyphal growth. The yeast form is non-pathogenic and infected corn has been known as a delicacy in Mexico for centuries, showing *U. maydis* to be safe for human consumption. This basidiomycete fungus serves as an excellent model system not only for plant pathogenicity, but also for cell and RNA biology (Steinberg and Perez-Martin, 2008; Béthune et al., 2019).

Besides its role as a model system, *U. maydis* is currently being developed as a production chassis for a wide range of biotechnological relevant compounds. This includes itaconic acid as a chemical platform molecule for biofuels, ustilagic acid and mannosylerythritol lipids (MEL) as biosurfactants, and various antibody formats as valuable proteins (Teichmann et al., 2010; Feldbrügge et al., 2013; Sarkari et al., 2014; Terfrüchte et al., 2017; Becker et al., 2020). Furthermore, strains were generated to utilise cellobiose, xylan and polygalacturonic acid as a carbon source in the yeast phase, so that plant cell wall components including pectin can be used as starting point for sustainable production (Geiser et al., 2016; Müller et al., 2018; Stoffels et al., 2020).

*U. maydis* is highly amenable for genetic engineering. Stable strains can be generated by homologous recombination. A comprehensive molecular toolbox, including inducible promoters, fluorescence reporters and epitope tags, is available (Brachmann et al., 2004; Terfrüchte et al., 2014). Here, we add the 2A peptide technology to the growing list of molecular tools in order to further increase the methods spectrum for synthetic biological approaches and biotechnology.

## Results and Discussion

### Establishing a Reporter System for Screening the Activity of 2A Peptides

**FIGURE 1:**
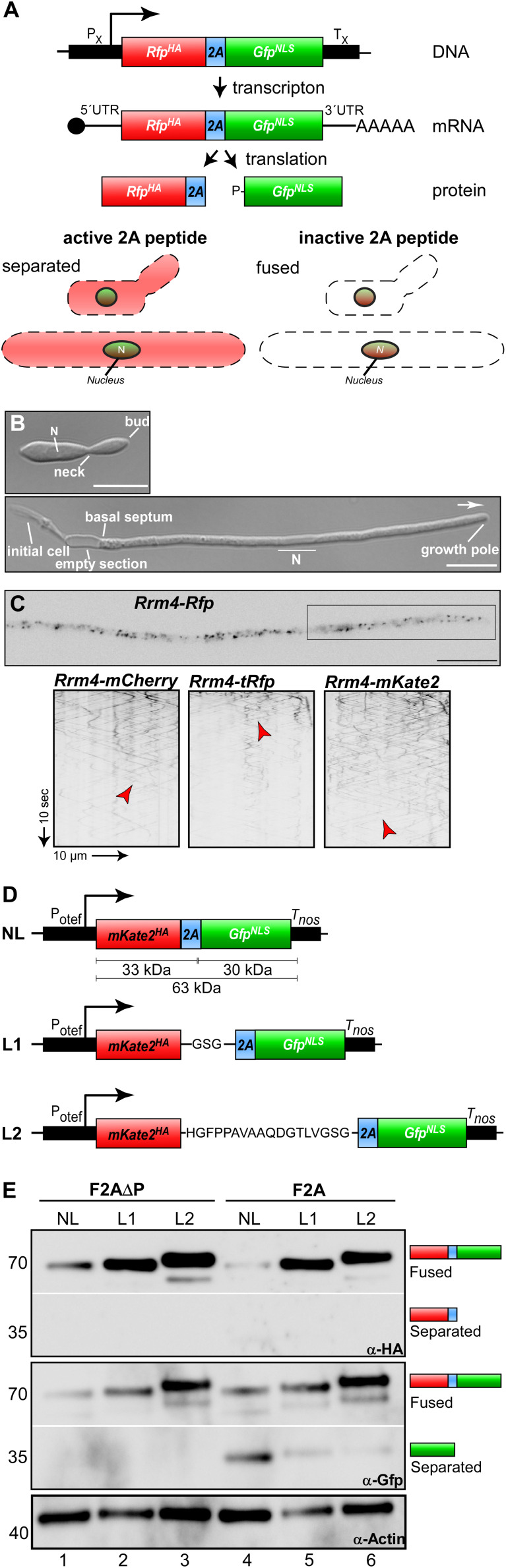
Reporter system for screening the activity of 2A peptides. **(A)** Top: Schematic representation of bi-cistronic reporter: constitutively active promoter (P_X_), ORF encoding a red fluorescent protein fused to a HA tag (red, RFP^HA^), 2A peptide of interest (blue) and green fluorescent protein fused to a nuclear localization sequence (green, GFP^NLS^), transcriptional terminator (T_X_). The bi-cistronic mRNA is indicated with 5′ cap structure (filled circle), 5′ and 3′ untranslated region (5′ UTR and 3′ UTR, respectively), and poly(A) tail (AAA). Bottom: Scheme of fluorescent protein localization within yeast and hyphal cell of *U. maydis* showing an active 2A peptide (left) or an inactive version (right). **(B)** Growth of laboratory strain AB33 (yeast and hyphae at the top and bottom, respectively). Unipolar growing hypha (6 h.p.i.; growth direction is indicated with an arrow; N, nucleus; scale bar 10 μm). **(C)** Top: Localization of Rrm4-Rfp within hypha (6 h.p.i.; inverted fluorescence image; scale bar 10 μm). Rectangle indicates region where kymographs were recorded. Bottom: Kymographs of AB33 hyphae (6 h.p.i.) expressing different Rrm4 fusions (arrow length on the left and bottom indicates time and distance). Bidirectional movement is indicated with red arrowheads. **(D)** Schematic representation of linker constructs for 2A peptide analysis. Constitutively active promoter (P_otef_), mKate2 fused to a HA tag (red), F2A (blue), Gfp fused to NLS (green), transcriptional terminator (T_nos_); no linker (NL), GSG linker (L1), 18 aa long linker (L2). **(E)** Western blot analysis shows ratio of fused to separated proteins (antibodies are given at the bottom, size of marker proteins in kDa at the left).

To test the activity of different 2A peptides in *U. maydis*, we designed a bi-cistronic reporter system consisting of the following components (Figure 1A): (i) constitutively active promoter, (ii) upstream ORF encoding a red fluorescent protein, (iii) 2A peptide of interest, (iv) downstream ORF encoding a green fluorescent protein fused to a nuclear localisation signal (NLS) and (v) heterologous transcriptional terminator. Thus, an active 2A peptide would result in increased cytoplasmic red fluorescence while green fluorescence will be located in the nucleus. This enables *in vivo* evaluation of the separation activity (Figure 1A; see below).

As a first step we tested different red fluorescent proteins. Currently, the monomeric mCherry protein from *Discosoma* sea anemones is used in *U. maydis* (Baumann et al., 2014). However, the protein exhibits fast photobleaching and its pH stability results in strong fluorescence in vacuoles. This causes difficulties in quantification and localisation of cognate fusion proteins. Therefore, we selected two additional versions, TagRFP and mKate2, both derived from the sea anemone *Entacmaea quadricolor* (Shcherbo et al., 2007; Shcherbo et al., 2009). For evaluation, we generated C-terminal fusions with the RNA-binding protein Rrm4. During hyphal growth, this posttranscriptional regulator links cargo mRNAs to transport endosomes for their long-distance transport along microtubules. Thereby, Rrm4 orchestrates endosome-coupled translation during transport (König et al., 2009; Baumann et al., 2014; Béthune et al., 2019; Olgeiser et al., 2019). Membrane-coupled translation is a wide spread mechanism and endosome-coupled translation was recently also found in neurons (Cioni et al., 2019; Liao et al., 2019).

The correct subcellular localisation of Rrm4 is intensively studied and can easily be scored during hyphal growth because it shuttles on almost all transport endosomes (Figures 1B,C; Baumann et al., 2012; Pohlmann et al., 2015). To generate the fusion proteins, the heterologous ORFs were synthesised according to a context-dependent codon usage that prevents premature poly(A) adenylation of foreign sequences in *U. maydis* (Zarnack et al., 2006; Zhou et al., 2018)^1^. Corresponding constructs were inserted at the *rrm4* locus to generate translational fusions in the genetic background of AB33 by homologous recombination (see section “Materials and Methods”). AB33 is genetically modified to allow an efficient and highly synchronous switch between yeast and hyphal growth by changing the nitrogen source of the medium. Hyphae expand at the growing tip and insert basal septa resulting in the formation of regularly spaced empty sections (Figure 1B; Brachmann et al., 2001).

All three Rrm4 fusion proteins were fully functional and direct comparison revealed that TagRFP exhibits the highest fluorescence intensity. However, mKate2 is clearly more photostable than the other two fluorescent proteins allowing detailed analyses of subcellular localisation over an extended period of time (Figure 1C). Therefore, we chose mKate2 in our system (Figures 1D,E) and recommend its application in live cell imaging in *U. maydis*.

The respective transcript encoding mKate2^HA^-2A-GFP^NLS^ (eGFP, enhanced version of GFP, Clontech) was expressed under the control of the constitutively active promoter P_otef_ (Figure 1D; Spellig et al., 1994). The constructs carried a nourseothricin resistance cassette and were targeted to the *upp3* locus of the laboratory strain AB33*upp3*Δ by homologous recombination. *upp3* was deleted using a hygromycin resistance cassette. It encodes a secreted protease that is dispensable for viability (Sarkari et al., 2014; Supplementary Tables S1, S2). This targeting strategy was advantageous, since all constructs were positioned at the identical genomic locus. Furthermore, counter-selection (i.e., loss of hygromycin resistance and gain of nourseothricin resistance) allowed fast pre-screening of homologous recombination events (see section “Materials and Methods”). Since it had previously been mentioned that linker sequences influence the expression of open reading frames connected by 2A peptides (Holst et al., 2006; Gao et al., 2012; Souza-Moreira et al., 2018), we tested different linker versions. We compared assemblies without linker (no linker; NL) with those comprising a short GSG linker sequence (L1) and a longer version of 18 amino acids (L2). We started comparing the linker sequences with the canonical F2A sequence from FMDV. As a control we expressed the same sequence without the essential C-terminal proline (F2AΔP; Ryan et al., 1991). As mentioned above all heterologous sequences were designed according to the context-dependent codon usage for *U. maydis* (Zarnack et al., 2006; Zhou et al., 2018; see text footnote 1).

To assess separation efficiency, yeast cells were grown in complete medium and harvested during exponential growth phase. Total protein extracts were tested by Western blot analysis using commercially available α-HA and α-Gfp antibodies (Figure 1E; see section “Materials and Methods”). Importantly, comparing constructs without linker to those with linkers L1 or L2, we observed that the absence of a linker resulted in low expression levels (Figure 1E, lane 1 and 4) and the expression level increases with the length of the linker sequence. We observed weak F2A ORF separation activity for all constructs that included the F2A peptide sequence (Figure 1E, lane 4–6), unless the C-terminal proline was deleted (Figure 1E, lane 1–3). Based on these results we chose the linker sequence L2 for further experiments. In essence, we succeeded in setting-up a straightforward reporter system demonstrating that in accordance with earlier reports, proline is essential for activity (Sharma et al., 2012) and linker sequences improve the expression of 2A peptide-containing transcripts (Holst et al., 2006; Gao et al., 2012; Souza-Moreira et al., 2018).

### Screening the Activity of Various 2A Peptides in *U. maydis*

**FIGURE 2:**
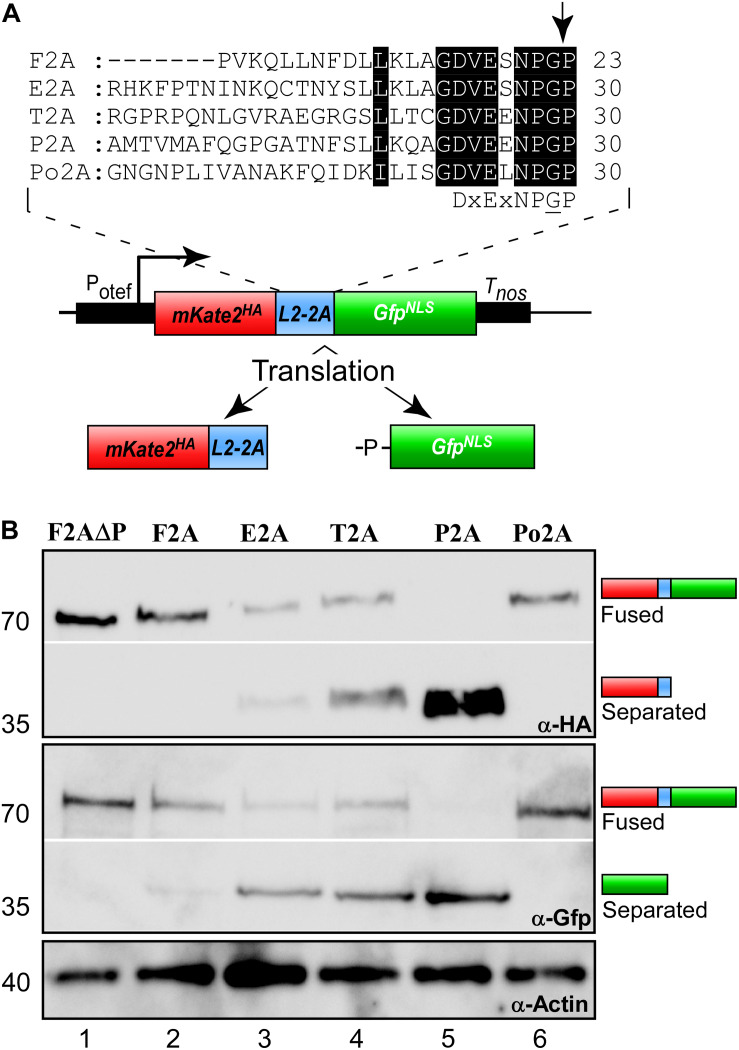
Separation efficiencies of 2A peptides. **(A)** Top: Sequence comparison of 2A peptides (highlighted in black: conserved and important amino acids; arrow indicates point of peptide separation; consensus sequence below). Bottom: Schematic representation of reporter construct and the resulting proteins after translation due to 2A peptide separation: Constitutively active promoter (P_otef_), mKate2 fused to a HA tag (red), L2 linker upstream of the 2A peptide of interest (blue), Gfp fused to NLS (green), transcriptional terminator (T_nos_). **(B)** Western blot analysis showing ratio of fused to separated polypeptides for the different tested 2A peptides (antibodies are given at the bottom, size of marker proteins in kDa at the left).

Previously it has been shown that 2A peptides from various viruses exhibited different activities in the ascomycete *Saccharomyces cerevisiae* (Souza-Moreira et al., 2018) as well as in mammalian cells (Kim et al., 2011). In order to identify suitable 2A peptides for *U. maydis*, we selected five versions: four 2A peptides with different separation activities reported from other systems (Table 1 and Figure 2A): P2A, Porcine teschovirus-1 (PTV); T2A, *Thosea asigna* virus (TaV); E2A, *Equine rhinitis* A virus (ERAV); F2A, Foot-and-mouth-disease virus (FMDV). In addition, we chose Po2A from porcine rotavirus (PoRV) C that was not previously studied. Sequences were inserted downstream of the L2 linker (Figure 2A) and constructs were again targeted to the *upp3* locus of AB33*upp3*Δ. To assess the different 2A peptide activities, yeast cells were grown in complete medium and total protein extracts analysed by Western blot experiments (see above). The activities were deduced qualitatively from the ratio of fused versus separated forms of mKate2^HA^ and Gfp^NLS^. We observed that F2A and Po2A were hardly functional (Figure 2B, lane 2 and 6). There is a clear difference between E2A, T2A and P2A, and the latter showed the highest activity (Figure 2B, lane 3–5).

**TABLE 1:** Comparison of 2A peptide separation efficiencies in different organisms.

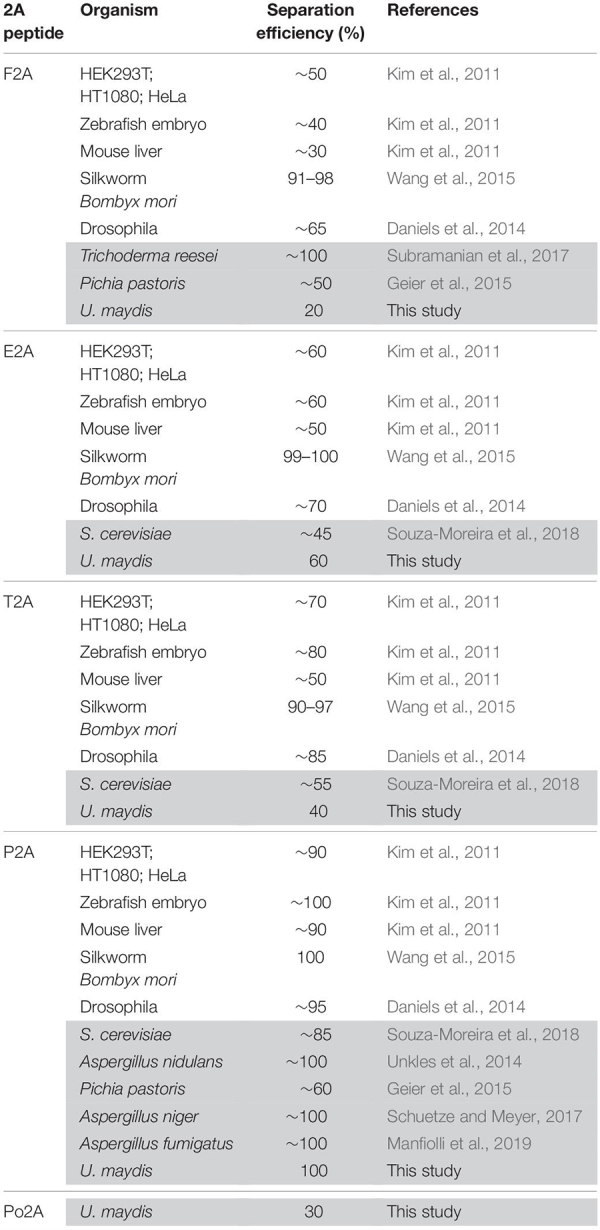

*Fungal organisms shaded in gray.*

Next, we studied the separation activity *in vivo*. To this end, we made use of the fact that products localise differently after separation. The separated mKate2^HA^ is expected to localise in the cytoplasm in contrast to GFP^NLS^, which should mainly localise to the nucleus (Figure 1A). A similar set-up with two fluorescence proteins as reporters has successfully been used before to study separation in mammalian cells, silkworm and *S. cerevisiae* (Wang et al., 2015; Liu et al., 2017). In *Aspergillus nidulans*, the activity of 2A peptides was recorded using split Yfp subunits. Both halves of the fluorescence protein carried an NLS to direct them to the nucleus for interaction. Hence, expression of the corresponding polycistronic mRNA resulted in nuclear fluorescence (Hoefgen et al., 2018). In our set-up, we fused the NLS to the Gfp reporter (de Felipe and Ryan, 2004; Provost et al., 2007; Kim et al., 2011).

**FIGURE 3:**
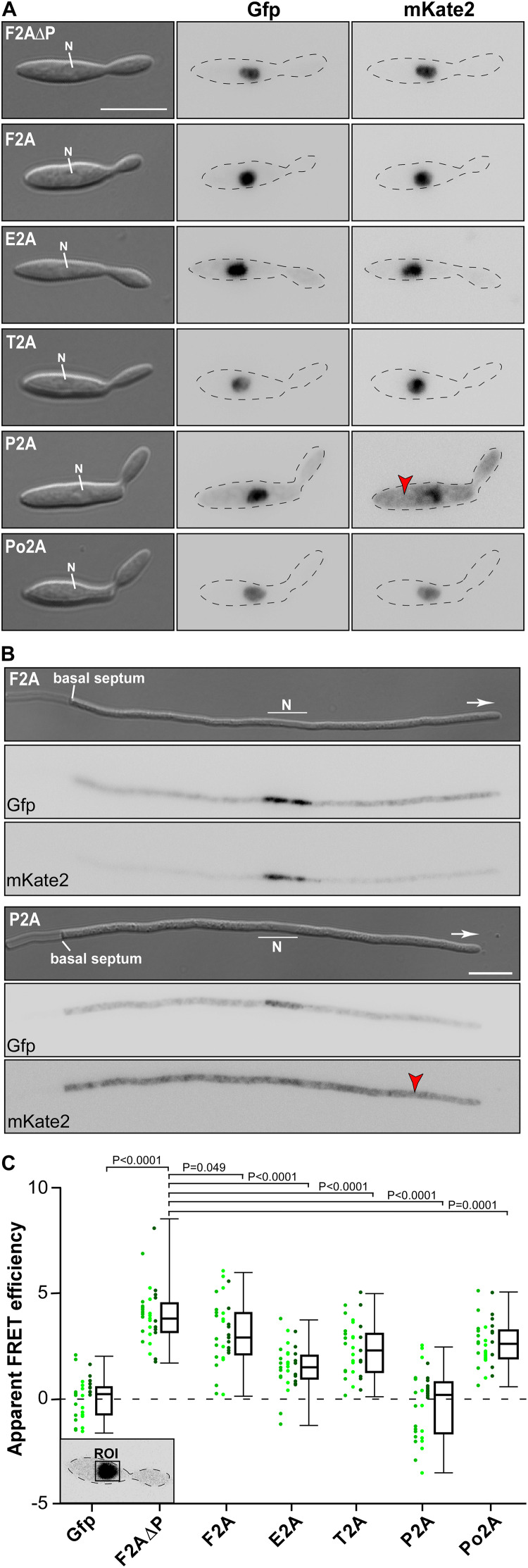
Separation efficiencies of 2A peptides analysed *in vivo*. Morphology and fluorescence microscopy of yeast **(A)** and hyphal **(B)** cells expressing reporter construct for analysis of 2A peptide separation efficiency (inverted fluorescence micrograph; N, nucleus; strong fluorescence signal in cytoplasm is indicated by red arrowhead; scale bar 10 μm; for hyphal cells: 6 h.p.i.; growth direction is indicated by arrow). **(C)** Apparent FRET efficiencies of tested 2A peptides determined within nucleus (ROI, region of interest; green dots show apparent FRET efficiencies in individually measured cells; each shade of green represents an independent experiment, *n* = 3; box and whisker plot shows median and min and max values; for each experiment >10 yeast cells were analysed; unpaired, two-tailed Mann-Whitney test was used).

Studying yeast cells revealed that the 2A peptide at the N- and C-terminus did not interfere with the fluorescence of mKate2 or the HA epitope tag. Comparing the 2A peptides we observed that only in the case of P2A we detected strong cytoplasmic red fluorescence, indicating that this 2A peptide seems to exhibit high separation efficiency (Figure 3A). Performing identical analyses in hyphae of *U. maydis* showed the same tendency (Figure 3B and Supplementary Figure S1). Hence, with our set-up we could easily test different stages of the fungal life cycle verifying that there is no developmental regulation of 2A peptide activity. In both cases P2A was the most promising candidate.

Finally, we assessed the efficiency of the separation activity of the five different 2A peptides *in vivo* utilizing the read-out of fluorescence resonance energy transfer (FRET) between the reporters Gfp and mKate2 (Szymczak et al., 2004; characterisation of Gfp and mKate2^2^). The phenomenon of FRET can only occur if the donor fluorophore (Gfp^NLS^) is in very close proximity (below 10 nm) to the acceptor (mKate2^HA^). The further the two fluorescence proteins are separated from each other the less FRET is detectable. Thus, FRET experiments conducted in the nucleus reveal the proportion of reporter proteins that are not separated (Figure 3C), because the unseparated reporter fusion proteins mKate^HA^-2A-Gfp^NLS^ would accumulate in this compartment due to the presence of the NLS. Note that mKate^HA^ is able to enter the nucleus due to its small size even in the absence of an NLS (Figure 3B). However, a so called “bystander FRET” effect (unspecific FRET due to crowding of non-interacting donor and acceptor) is only expected at very high concentrations of proteins. Thus, a small amount of free mKate2 protein as observed in this case does not interfere with the measurement. The experimental set-up revealed different low-level, but very stable FRET effects in the investigated 2A samples. The highest apparent FRET efficiency was observed in the nucleus of cells expressing the unseparated negative control F2AΔP, contrary to reduced FRET_app_ efficiencies within cells containing the assemblies with the different 2A peptides (Figure 3C). This underlines the sensitivity of the FRET measurements.

The experimental set-up was sensitive enough to detect the low F2A activity *in vivo* (Figure 3C). P2A shows FRET rates, which were comparable to a control strain only expressing Gfp, emphasizing a nearly 100% separation rate determined *in vivo*. The negative value of FRET_app_ is due to slight acquisition bleaching of Gfp. This is consistent with the results indicated by the Western blot analysis (Figure 2B) and the live cell fluorescence microscopy (Figures 3A,B). In essence, using a sophisticated *in vivo* strategy we were able to show that P2A exhibits the highest separation efficiency for *U. maydis*. Thus, we used P2A for further applications. When comparing different organisms, it is evident that 2A peptides exhibit a wide range of activities (Table 1). This underlines the importance of testing various 2A peptides regarding their separation efficiency, although P2A works best in most systems tested so far.

### Applying 2A Peptide Technology to Monitor Regulated Gene Expression Over Time

**FIGURE 4:**
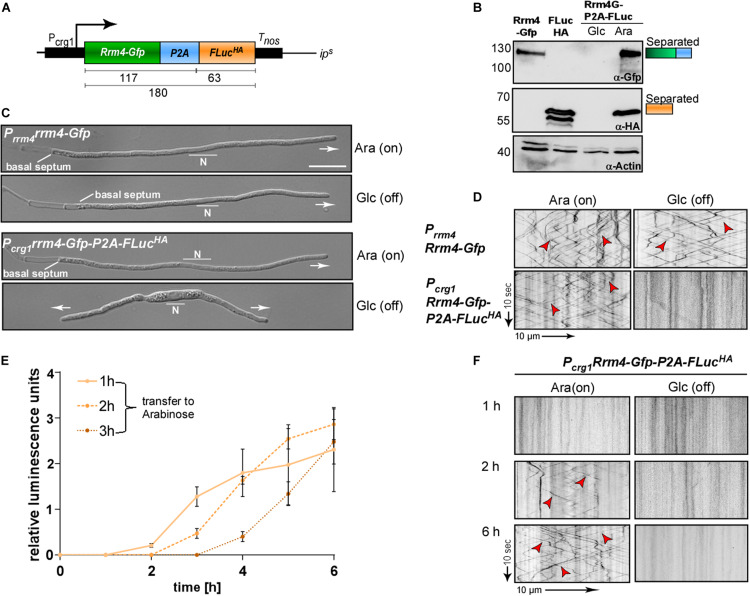
Following regulated gene expression over time using peptide P2A. **(A)** Schematic representation of a construct to analyse regulated gene expression (expected size of proteins in kDa given below). Arabinose inducible promoter (P_crg1_), Rrm4 fused to Gfp (green), L2 linker fused to P2A (blue), Firefly luciferase (FLuc) fused to HA tag (orange), transcriptional terminator (T_nos_). **(B)** Western blot analysis of AB33 derivates under induced and uninduced conditions (Glc: Glucose; Ara: Arabinose; antibodies are given at the bottom, size of marker proteins in kDa at the left). **(C)** Morphology of growing hypha expressing Rrm4 and FLuc separated by P2A in induced and uninduced conditions (6 h.p.i.; Glc: Glucose, Ara: Arabinose; growth direction is indicated by arrow; N, nucleus; scale bar 10 μm). **(D)** Kymographs of AB33 hyphae (6 h.p.i.) expressing Rrm4 and FLuc separated by P2A in induced and uninduced conditions (Glc: Glucose; Ara: Arabinose; arrow length on the left and bottom indicates time and distance). Bidirectional movement is indicated with red arrowheads. **(E)** Luciferase activity determination of strains expressing Rrm4-Gfp-L2-P2A-FLuc^HA^ shifted to hyphal growth at time point 0. After 1, 2 or 3 h cells were transferred to arabinose-containing medium (error bars, SEM; *n* = 3 independent experiments, relative luminescence units are given. **(F)** Kymographs of hypha carrying construct Rrm4-Gfp-P2A-FLuc after different time points of switching to arabinose containing medium (Glc: Glucose; Ara: Arabinose; arrow length on the left and bottom indicates time and distance; bidirectional movement is indicated with red arrowheads).

To illustrate applicability, we designed a strategy for an efficient read-out for monitoring regulated gene expression. We aimed to quantify induction of a promoter over time using straightforward reporter enzyme activity. To this end, we combined the *Photinus pyralis* Firefly luciferase (FLuc; Hüsemann et al., in preparation) with Rrm4-Gfp on a bi-cistronic mRNA using the P2A peptide sequence and the L2 linker (Figure 4A). In this proof-of-concept study, we expressed a Gfp fusion protein, so that *rrm4* expression can be easily monitored *in vivo* using live cell imaging. In future applications the 2A peptide technology will be used to follow regulated expression of untagged proteins over time.

A luciferase was successfully used before to determine P2A activity in *Aspergillus niger* (Schuetze and Meyer, 2017). The activity of Firefly luciferase can easily be detected by adding the substrate luciferin to the cells. As an example for regulated expression, we employed the promoter P_crg__1_, which is active in the presence of arabinose and inactive in the sole presence of glucose (Figure 4A; Brachmann et al., 2001). The construct was integrated at the *ip*^*S*^ locus by homologous recombination in the genetic background of AB33rrm4Δ (Loubradou et al., 2001; Materials and Methods). Western blot experiments of hyphae growing for 6 h under uninduced and induced conditions revealed that the luciferase as well as Rrm4-Gfp were expressed in arabinose-containing medium and, as expected, both were fully separated (Figure 4B and Supplementary Figure S2). Analysing hyphal growth in glucose and arabinose revealed that only in glucose-containing medium hyphae grew in a bipolar mode; this aberrant growth form is characteristic for loss of Rrm4 (Figure 4C; Becht et al., 2006). In medium containing arabinose, however, the cells grew unipolarly as expected (Figure 4C). Studying dynamic subcellular localisation demonstrated that endosomal shuttling of Rrm4-Gfp was not influenced by the carbon source (arabinose or glucose) in the control strain expressing Rrm4-Gfp at the native locus (AB33P_rrm__4_Rrm4-Gfp). However, no fluorescence signal of Rrm4-Gfp was detected in the presence of glucose (Figure 4D), indicating that the promoter is switched off.

To analyse the induction of the P_crg__1_ promoter in the presence of arabinose, hyphal growth was elicited in the presence of glucose. At different times the cells were transferred into arabinose-containing medium and FLuc activity as well as the fluorescence signal of Rrm4-Gfp were determined. Luciferase activity increased after shifting to arabinose-containing medium indicating activation of the P_crg__1_ promoter (Figure 4E). Consistently, dynamic live cell imaging showed that endosomal shuttling of Rrm4-Gfp was detectable after 2 h of growth in arabinose-containing medium (Figure 4F). After 6 h the signal intensities were comparable to a strain expressing Rrm4-Gfp under the control of the native promoter. Thus, using 2A peptide technology allows simple and reliable quantification of gene expression. This can be used to study other aspects like mRNA stability, protein turnover or degradation, as well as to monitor the expression of certain mRNAs *in planta*.

### Synthetic Constitutive Co-expression of MEL Cluster Enzymes With the P2A Peptide

**FIGURE 5:**
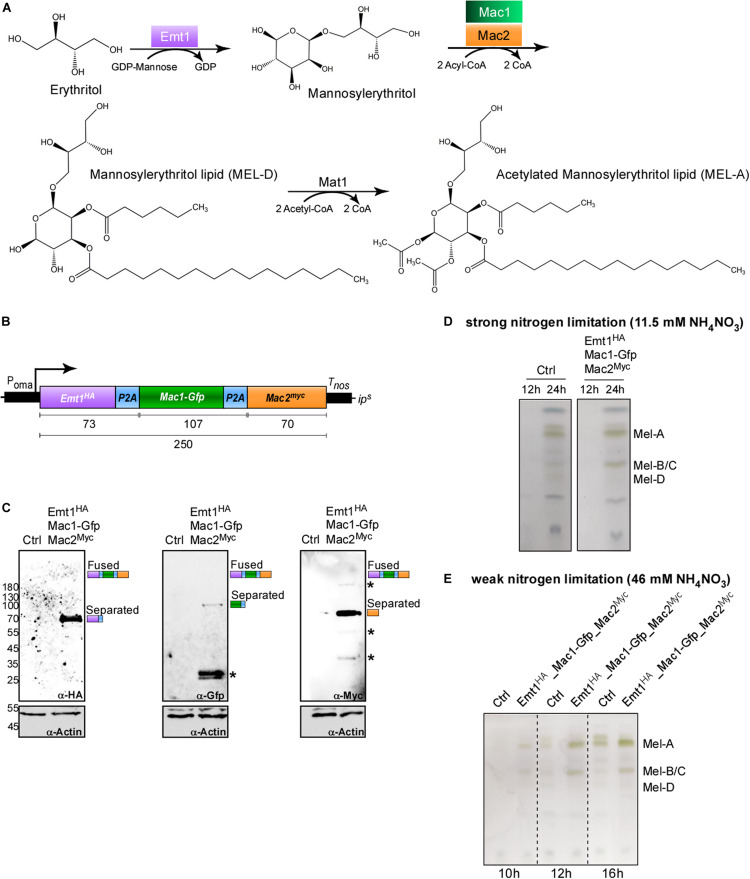
Usage of P2A enables production of biosurfactants. **(A)** Metabolic MEL synthesis pathway in *U. maydis*. GDP-mannose is added to an erythritol moiety by the mannosyl transferase Emt1 (purple) with consumption of GDP, resulting in the formation of mannosylerythritol. C_2_ and C_3_ positions are acylated with C_2_-C_18_ fatty acid chains. This step is catalyzed by the acyl transferases Mac1 (green) and Mac2 (orange) and results in the formation of the deacetylated variant MEL-D. MEL-D is acetylated in positions C_4_ and C_6_ by the acetyl transferase Mat1, resulting in the formation of MEL-A (C_4_ and C_6_ acetylated), MEL-B (C_6_ acetylated, not shown) and MEL-C (C_4_ acetylated, not shown). **(B)** Schematic representation of tri-cistronic expression construct for MEL-D production using cytoplasmic versions of Mac1 and Mac2: constitutively active promoter (P_oma_), ORF encoding Emt1 fused to HA tag (purple, Emt1^HA^), L2 fused to P2A (blue, P2A), Mac1 fused to Gfp (green, Mac1-Gfp), L2 fused to P2A (blue, P2A) Mac2 fused to a myc tag (orange, Mac2^*myc*^), transcriptional terminator (T_nos_). **(C)** Western blot analysis of protein synthesis via tri-cistronic mRNA (antibodies are given at the bottom, size of marker proteins in kDa at the left, asterisks indicate protein degradation). **(D)** Thin layer chromatography of glycolipids from whole cells under conditions of strong nitrogen limitation. **(E)** Thin layer chromatography of glycolipids from whole cells under conditions of weak nitrogen limitation (progenitor strain MB215rua1Δ, Ctrl).

To produce biosurfactants, genes for several biosynthetic enzymes need to be expressed simultaneously. This occurs in wild type strains, when the nitrogen source is limited. Here, we tested the applicability of the 2A peptide for synthetic activation of a secondary metabolite gene cluster in a biotechnological approach. This offers the clear advantage that synthesis can be uncoupled from nitrogen metabolism. *U. maydis* is a natural producer of the glycolipids mannosylerythritol lipid (MEL) and Ustilagic acid (UA). The biosynthetic enzymes for MEL production are encoded in a gene cluster activated upon nitrogen starvation (Figure 5A; Hewald et al., 2006). To achieve synthetic activation from a strong constitutively active promoter (P_oma_), we designed a tri-cistronic messenger RNA with three enzymes of the pathway. We used P2A combined with the L2 linker, and integrated the construct at the *ip*^*s*^ locus of the glycolipid producing strain MB215 lacking biosynthesis of UA (MB215rua1Δ; Ctrl, Figure 5B). Genes for the mannosyltransferase Emt1 and cytoplasmic versions of the acyl transferases Mac1 and Mac2 (Freitag et al., 2014) were encoded on a single mRNA. For protein detection different sequences for protein tags were used (Figure 5B). Western blot experiments confirmed production of the three enzymes as separated proteins, indicating that a tri-cistronic construct is also functional (Figure 5C). In control experiments, we analysed the glycolipid profile using thin-layer chromatography under strong nitrogen limitation. As expected, MEL variants were detectable after 24 h. The presence of the tri-cistronic construct did not alter MEL synthesis (Figure 5D). However, studying the glycolipid profile under weak nitrogen limitation resulted in the production of MEL variants already detectable after 10 h of growth in strains expressing the tri-cistronic mRNA (Figure 5E; see section “Materials and Methods”). The progenitor strain lacking this construct was only able to produce MELs at much later time points (Figure 5E). Thus, using the 2A peptide technology we succeeded to express three biosynthetic enzymes from a single mRNA in order to produce MELs efficiently under conditions of weak nitrogen limitation.

### Conclusion

The strategy to use 2A peptides to co-express multiple genes of biosynthetic pathways has successfully been used before in ascomycetes like *S. cerevisiae* and different *Aspergillus* species (Beekwilder et al., 2014; Unkles et al., 2014; Hoefgen et al., 2018; Schuetze and Meyer, 2017). This approach has now been expanded to the basidiomycete *U. maydis* underlining the broad applicability of polycistronic mRNAs in biotechnology. Here we present a straightforward strategy to analyse and quantitatively assess the functionality of 2A peptides *in vivo*. The analysis was conducted with five different versions but can easily be extended to other 2A peptides. The initial fluorescence readout of cytoplasmic red fluorescence is very simple and the FRET approach allows a sensitive and quantitative measurement of the separation activity. We successfully applied the best performing P2A peptide in basic and applied science demonstrating its efficient performance. With this proof-of-principle in hand, numerous new future applications like defined co-expression of subunits of protein complexes as well as efficient expression of heterologous biosynthetic gene clusters are conceivable.

## Materials and Methods

### Plasmids, Strains and Growth Conditions

For cloning of plasmids, *E. coli* Top10 cells (Life Technologies, Carlsbad, CA, United States) were used. Transformation, cultivation and plasmid isolation were performed using standard techniques. *U. maydis* strains either derive from the lab strain AB33, in which the hyphal growth can be induced (Brachmann et al., 2001) or from the wild type strain MB215. AB33: Yeast-like cells were grown in complete medium (CM) supplemented with 1% glucose. Hyphal growth was induced by switching the nitrogen source by changing the media to nitrate minimal medium (NM) supplemented with 1% glucose or arabinose. MB215: MEL production cultures were incubated in unbaffled 300 ml flasks in 20 ml Verduyn-C mineral medium (11.5 mM NH_4_NO_3_ (strong limitation) or 46 mM NH_4_NO_3_ (weak limitation), 0.28 M (5%) glucose, 0.1 M MES pH 6.5, 3.6 mM KH_2_PO_4_, 0.8 mM MgSO_4_^∗^7H_2_O, 51 μM EDTA, 37 μM FeCL_3_^∗^6H_2_O, 16 μM H_3_BO_3_, 15.6 μM ZnSO_4_^∗^7H_2_O, 6.7 μM MnCl_2_^∗^2H_2_O, 2.3 μM CoCl_2_^∗^6H_2_O, 1.9 μM Na_2_MoO_4_^∗^2H_2_O, 0.6 μM KI) at 300 rpm and 28°C. Cultures were inoculated to an OD_600_ of 0.1 and cultivated for up to 24 h.

Detailed growth conditions and cloning strategies for *U. maydis* are described elsewhere (Brachmann et al., 2004; Baumann et al., 2012; Terfrüchte et al., 2014; Beyer et al., 2015). All plasmids were sequenced to verify correctness. *U. maydis* strains were generated via homologous recombination within the *ip*^*S*^ or the *upp3* locus (Loubradou et al., 2001) by transforming progenitor strains with linearised plasmids, except for MB215rua1Δ that was obtained with CRISPR-Cas technology (Schuster et al., 2016). Correctness of the strains was verified by counter selection (where possible), analytic PCR and Southern Blot analysis (Brachmann et al., 2004). A description of all plasmids and strains is summarised in Supplementary Tables S1–S3. Sequences are available upon request.

### Microscopy, FRET and Image Analysis

For microscopy, yeast-like cells were grown for 12 h in complete medium. Microscopy was performed as described before (Baumann et al., 2012). The wide-field microscope Zeiss (Oberkochen, Germany) Axio Observer.Z1 provided with an Orca Flash4.0 camera (Hamamatsu, Japan) and objective lens Plan Apochromat (63×, NA 1.4) was used. Excitation of fluorescently labelled proteins was carried out using a laser-based epifluorescence-microscopy. A VS-LMS4 Laser Merge-System (Visitron Systems, Puchheim, Germany) combines solid state lasers for the excitation of Gfp (488 nm/100 mW) and Rfp/mCherry (561 nm/150 mW). All modules of the microscope systems were controlled by the software package VisiView (Visitron). This was also used for image processing.

FRET-APB was measured using a Zeiss LSM780 laser-scanning microscope and a C-Apochromat 40×/1.20 korr M27 water objective (Carl Zeiss, Jena, Germany). GFP was excited with a 488 nm argon laser at an output power of 0.3% and emission of the fluorescence signal was detected between 490 and 552 nm using a 32 channel GaAsP detection unit. mKate2 was excited using a 561 nm DPSS laser at an output power of 5% and emission detected between 588 and 686 nm. In total, a time series of 20 frames (256 times 256 pixels) at a pixel time of 3.15 μs/pixel was recorded with no line averaging. After the 5th frame, the nucleus and the surrounding area of yeast-like cells was bleached at 100% laser power of the 561 nm laser, for 50–100 iterations. After the bleaching, 15 more frames were recorded. The “apparent FRET efficiency,” FRET_app_ was determined by comparing the fluorescence intensity in the bleached “region of interest” (ROI) of the donor fluorophore after bleaching of the acceptor fluorophore mKate2 according to the formula:

$${\text{FRET}}_{\text{app}}=\frac{{\text{Intensity}}_{\mathrm{Donor}\,\mathrm{after}}-{\text{Intensity}}_{\mathrm{Donor}\,\mathrm{before}}}{{\mathrm{Intesity}}_{\mathrm{Donor}\,\mathrm{after}}}\times 100$$

### Protein Extracts and Western Blot Analysis

*U. maydis* yeast-like cells or hyphae (6 h.p.i) were harvested by centrifugation (7,546 *g*, 10 min) and resuspended in 1 ml urea buffer (8M urea, 50 mM Tris/HCl pH8) to which protease inhibitors were freshly added (1 tablet of Complete protease inhibitor per 25 ml, Roche, Mannheim, Germany; 1 mM DTT; 0.1 M PMSF; 0.5 M benzamidine). After adding 200 μl of glass beads the cells were disrupted in 1.5 ml Eppendorf tubes with the Mixer Mill MM400 (Retsch, Haan, Germany) by agitating for 10 min at 30 Hz at 4°C. For hyphae, the cells were agitated three times with cooling steps of 10 min in between. Protein concentrations were measured with the Bradford assay (Bio-Rad, Munich, Germany) and samples were adjusted to equal amounts. For Western Blot analysis, protein samples were supplemented with Laemmli buffer and heated to 95°C for 6 min followed by centrifugation for 30 s at 16,200 *g*. Proteins were separated by 8, 10, or 12% SDS-PAGE and transferred and immobilized in a nitrocellulose membrane (GE Healthcare, Munich, Germany) by semi-dry blotting. Proteins were detected using α-Gfp, α-HA (both Roche, Freiburg, Germany), α-Myc (Sigma-Aldrich Chemie GmbH, Munich, Germany) and α-Actin (MP Biomedicals, Eschwege, Germany) antibodies. As secondary antibody an anti-mouse IgG HRP conjugate (Promega, Madison, WI, United States) was used. Detection was carried out by using Amersham^TM^ ECL^TM^ Prime (GE Healthcare, Munich, Germany). The images were taken according to the manual’s instructions with a luminescence image analyser, LAS4000 (GE Healthcare, Solingen, Germany).

### Luminescence Measurements of Firefly Luciferase

To measure the luminescence of FLuc, 80 μl of cell suspension of hyphal growing cells was mixed with 20 μl of luciferin (20 mM Tricine, 2.67 mM MgSO_4_^∗^7H2O, 0.1 mM EDTA^∗^2H_2_O, 33.3 mM DTT, 524 μM ATP, 218 μM AcetylCoA, 131 μg/ml Luciferin, 5 mM NaOH, 264 μM MgCO_3_^∗^5H_2_O) in a white Berthold 96-well plate (Nr: 23300/23302). The measurements lasted 20 min and were conducted using a BertholdTech Mithras luminescence reader (Berthold Technologies, Bad Wildbad, Germany) with the driver version 1.07.

### MEL Extraction

MELs were sampled 10, 12, 16 and 24 h post inoculation. Therefore, 500 μl of whole cell culture broth were mixed with 500 μl of ethyl-acetate in 2 ml reaction tubes. MELs were extracted by shaking at 2,000 rpm for 15 min on a Vibrax VKA basic (IKA Werke GmbH & Co. KG, Staufen im Breisgau, Germany). Organic and aqueous phases were then separated by centrifugation at 21,100 *g* for 15 min. The organic phase was transferred into a fresh 1.5 ml reaction tube and evaporated at 70°C for 1 h. Dried MELs were resolved in 15 μl methanol.

### MEL Analysis by Thin-Layer Chromatography

MEL production was analysed by TLC using a two-chamber system (modified from Hewald et al., 2006). Glycolipid extracts of up to 15 μl were applied evenly onto half TLC silica plates (20 × 10 cm, Merck KGaA, Darmstadt, Germany). After drying, plates were placed into a TLC chamber saturated with 100 ml buffer I (65:25:4 chloroform, methanol, H_2_O) for 5 min. Afterward, plates were placed into a second TLC chamber saturated with 100 ml buffer II (9:1 chloroform, methanol) for 17 min. This step was repeated. For detection, dried TLC plates were sprayed with staining solution (50:1:0.5 glacial acetic acid, sulphuric acid, 4-methoxybenzaldehyde), dried again and incubated at 110°C for 5 min.

## Data Availability Statement

All datasets presented in this study are included in the article/Supplementary Material.

## Author Contributions

KM, MP, LH, NH, KS, MZ, and MF designed and planned the study. KM established the 2A peptides and analysed the promoter induction. MP optimized the production of the MELs. SW-P and KM performed the FRET analysis. KM, KS, and MF analysed the data. KM, KS, MZ, and MF designed and revised the manuscript. MF and KS directed the project. All authors contributed to the article and approved the submitted version.

## Conflict of Interest

The authors declare that the research was conducted in the absence of any commercial or financial relationships that could be construed as a potential conflict of interest.

## Footnotes

1 http://dicodon-optimization.appspot.com

2 https://www.fpbase.org/compare/egfp,mkate2
