# Supplementary material for: Establishing Polycistronic Expression in the Model Microorganism Ustilago maydis
Source: Front Microbiol. 2020 Jun 24;11:1384. doi: 10.3389/fmicb.2020.01384 (PMC7326815; doi:10.3389/fmicb.2020.01384)
Supplement: Supplementary file 1 [file Data_Sheet_1.pdf]

Supplementary Material of the manuscript entitled

Establishing polycistronic expression in the model microorganism *Ustilago maydis*

Kira Müntjes[1], Magnus Philipp[1], Lisa Hüsemann[2], Nicole Heucken[2], Stefanie Weidtkamp-Peters[3], Kerstin Schipper[1], Matias Zurbriggen[2] and Michael Feldbrügge[1, *]

Including Supplementary Figure S1-2 as well as Supplementary Table S1-S3

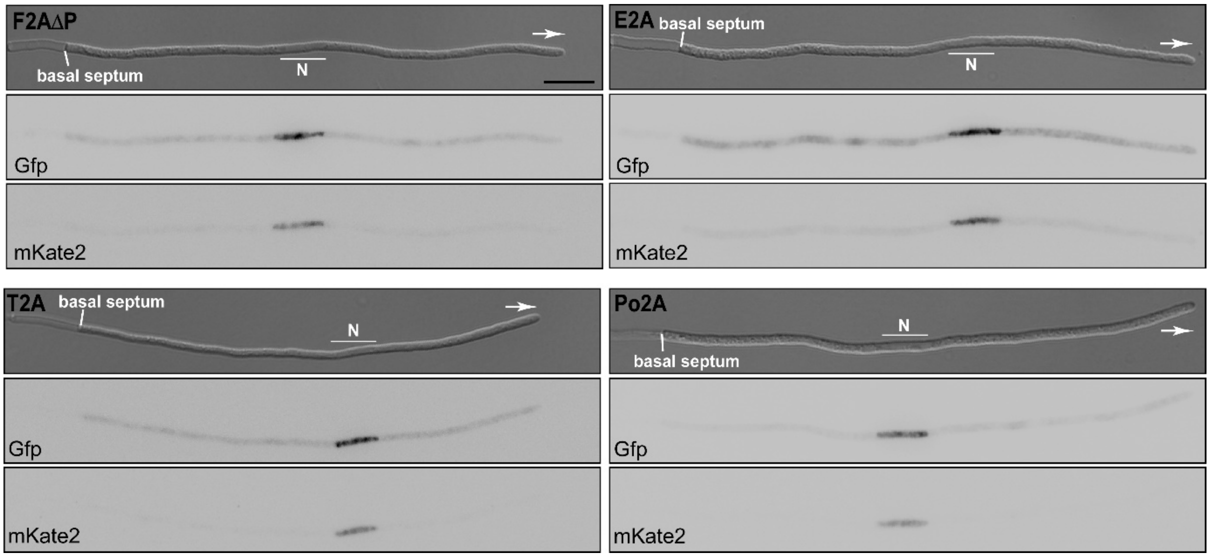

Supplementary Figure S1. Separation efficiency of 2A peptides during hyphal growth *in vivo.*
Hyphal cells (6 h.p.i.) expressing reporter construct mKate2^{HA}-L2-2A-Gfp^{NLS} (inverted fluorescence micrographs; N, nucleus; scale bar 10 µm; growth direction is indicated by arrow).

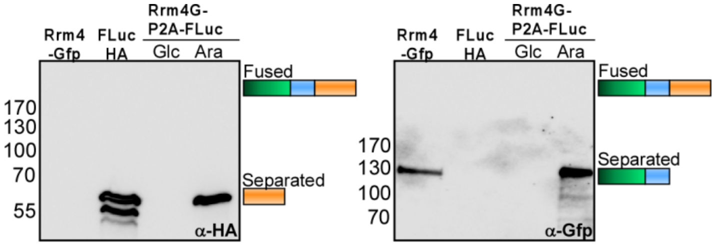

Supplementary Figure S2. Studying regulated gene expression using peptide P2A
Western blot analysis of AB33 derivates under induced and uninduced conditions (Glc: Glucose; Ara: Arabinose; antibodies are given at the bottom, size of marker proteins in kDa at the left).

Supplementary Table S1: *U. maydis* strains used in this study; UMa, internal reference number

Strain	Relevant genotype	Short description	Uma	Resistance cassette	Reference
**AB33**	a2 P*nar:bWebE1*	expression of active b heterodimer which is under the control of P_{nar1} promoter; hyphal growth can be induced by switching the nitrogen source	133		(Brachmann, 2001)
**AB33*rrm4*Δ**	*rrm4*Δ	carries a deletion of *rrm4*	273	HygR	(Becht et al., 2006)
**AB33Rrm4-Gfp**	*rrm4-Gfp*	expresses Rrm4 C-terminally fused to Gfp	274	NatR	(Becht et al., 2006)
**AB33Rrm4-mCherry**	*rrm4-mCherry*	expresses Rrm4 C-terminally fused to mCherry	830	HygR	(Baumann et al., 2014)
**AB33Rrm4-TagRfp**	*rrm4-TagRfp*	expresses Rrm4 C-terminally fused to TagRfp	1317	NatR	(Müntjes, 2015)
**AB33Rrm4-mKate2**	*rrm4-mKate2*	expresses Rrm4 C-terminally fused to mKate2	1985	NatR	This study
**AB33*upp3*Δ**	*upp3Δ*	carries a deletion of *upp3*	2148	HygR	This study
**AB33*upp3*Δ::P_{otef}-mKate2-HA-GSG-F2A-Gfp-NLS**	*mKate2-GSG-F2A-Gfp-NLS*	co-expresses mKate2 fused to a HA tag and Gfp fused to an NLS partial separated during translation by F2A	2495	NatR	This study
**AB33*upp3*Δ::P_{otef}-mKate2-HA-F2AΔP-Gfp-NLS**	*mKate2-F2AΔP-Gfp-NLS*	expresses a fusion protein of mKate2 fused to a HA tag and Gfp fused to an NLS; no separation during translation because of deletion of last proline of F2A	2520	NatR	This study
**MB215 rua1*Δ***	*rua1Δ (CRISPR)*	carries a deletion of *rua1*	2598	-	(Peter Stoffels, unpublished)
**AB33*upp3*Δ::P_{otef}-Firefly-HA**	*firefly-HA*	expresses Firefly luciferase fused to an HA tag	2644	NatR	(L. Hüsemann et al., in preparation)
**MB215*rua1Δ*/P_{oma}-*emt*1-HA-L2-P2A-Gfp-*mac*1-L2-P2A-*mac*2-3xmyc**	*emt1-HA-L2-P2A-gfp-mac1-L2-P2A-mac2-3xmyc*	co-expresses Emt1 fused to an HA tag, Mac1 N-terminally fused to Gfp and Mac2 fused to a triple myc tag; all proteins are separation by P2A fused N-terminally to L2 linker during translation	3084	CbxR	This study
**AB33*upp3*Δ::P_{otef}-mKate2-HA-L1-F2A-Gfp-NLS**	*mKate2-L1-F2A-Gfp-NLS*	co-expresses mKate2 fused to a HA tag and Gfp fused to an NLS; partial separated during translation by F2A fused N-terminally to L1 linker	3116	NatR	This study
**AB33*upp3*Δ::P_{otef}-mKate2-HA-L2-F2AΔP-Gfp-NLS**	*mKate2-L2-F2AΔp-Gfp-NLS*	expresses fusion of mKate2 fused to a HA tag and Gfp fused to an NLS; no separation during translation	3119	NatR	This study

		because of deletion of last proline of F2A			
**AB33*upp3*Δ::P_{otef}-mKate2-HA-L2-T2A-Gfp-NLS**	*mKate2-L2-T2A-Gfp-NLS*	co-expresses mKate2 fused to a HA tag and Gfp fused to an NLS partial separated during translation by T2A fused N-terminally to L2	3120	NatR	This study
**AB33*upp3*Δ::P_{otef}-mKate2-HA-L2-P2A-Gfp-NLS**	*mKate2-L2-P2A-Gfp-NLS*	co-expresses mKate2 fused to a HA tag and Gfp fused to an NLS separated during translation by P2A fused N-terminally to L2 linker	3123	NatR	This study
**AB33*upp3*Δ::P_{otef}-mKate2-HA-L2-F2A-eGFP-NLS**	*mKate2-L2-F2A-Gfp-NLS*	co-expresses mKate2 fused to a HA tag and Gfp fused to an NLS partial separated during translation by F2A fused to L2 linker	3144	NatR	This study
**AB33*upp3*Δ::P_{otef}-mKate2-HA-L2-Po2A-eGFP-NLS**	*mKate2-L2-Po2A-Gfp-NLS*	co-expresses mKate2 fused to a HA tag and Gfp fused to an NLS partial separated during translation by Po2A fused to L2 linker	3145	NatR	This study
**AB33*rrm4*Δ/Rrm4-Gfp-L2-P2A-Firefly-HA**	*rrm4-Gfp-L2-P2A-firefly-HA*	co-expresses Rrm4 fused C-terminally to Gfp and Firefly luciferase fused to HA tag; separation during translation through P2A N-terminally fused to L2	3212	CbxR	This study

Supplementary Table S2: *U. maydis* strains generated in this study;
UMa and pUMa, internal reference number

Strain	UMa	Relevant genotype	Transformed plasmid	Locus	Progenitor strain
**AB33Rrm4-mKate2**	1985	*Rrm4-mKate2*	P_{rrm4}-*rrm4*-mKate2 (pUMa2985)	*rrm4*	AB33*rrm4*Δ
**AB33*upp3*Δ**	2148	*upp3Δ*	p*upp3*Δ-hygR (pUMa1556)	*upp3*	AB33
**AB33*upp3*Δ::P_{otef}-mKate2-HA-F2A-Gfp-NLS**	2495	*mKate2-F2A-Gfp-NLS*	p*upp3*Δ::P_{otef}-mKate2-HA-F2A-Gfp-NLS (pUMa3407)	*upp3*	AB33*upp3*Δ
**AB33*upp3*Δ::P_{otef}-mKate2-HA-F2AΔP-Gfp-NLS**	2520	*mKate2-F2AΔP-Gfp-NLS*	p*upp3*Δ::P_{otef}-mKate2-HA-F2AΔP-Gfp-NLS (pUMa3435)	*upp3*	AB33*upp3*Δ
**MB215 *rua1Δ*/P_{oma}-*emt*1-HA-L2-P2A-Gfp-*mac*1-L2-P2A-*mac*2-3xmyc**	3084	*emt1-HA-L2-P2A-gfp-mac1-L2-P2A-mac2-3xmyc*	pip^R::P_{oma}-*emt*1-HA-L2-P2A-Gfp-*mac*1-L2-P2A-*mac*2-3xmyc (pUMa4131)	*ip^S*	MB215*rua1Δ*
**AB33*upp3*Δ::P_{otef}-mKate2-HA-L1-F2A-Gfp-NLS**	3116	*mKate2-L1-F2A-Gfp-NLS*	p*upp3*Δ::P_{otef}-mKate2-HA-L1-F2A-Gfp-NLS (pUMa4261)	*upp3*	AB33*upp3*Δ
**AB33*upp3*Δ::P_{otef}-mKate2-HA-L2-F2AΔp-Gfp-NLS**	3119	*mKate2-L2-F2AΔp-Gfp-NLS*	p*upp3*Δ::P_{otef}-mKate2-HA-L2-F2AΔP-Gfp-NLS (pUMa4197)	*upp3*	AB33*upp3*Δ
**AB33*upp3*Δ::P_{otef}-mKate2-HA-L2-T2A-Gfp-NLS**	3120	*mKate2-L2-T2A-Gfp-NLS*	p*upp3*Δ::P_{otef}-mKate2-HA-L2-T2A-Gfp-NLS (pUMa4202)	*upp3*	AB33*upp3*Δ
**AB33*upp3*Δ::P_{otef}-mKAte2-HA-L1-F2AΔp-Gfp-NLS**	3122	*mKate2-L1-F2AΔlastp-Gfp-NLS*	p*upp3*Δ::P_{otef}-mKate2-HA-L1-F2AΔP-Gfp-NLS (pUMa4273)	*upp3*	AB33*upp3*Δ
**AB33*upp3*Δ::P_{otef}-mKate2-HA-L2-P2A-Gfp-NLS**	3123	*mKate2-L2-P2A-Gfp-NLS*	p*upp3*Δ::P_{otef}-mKate2-HA-L2-P2A-Gfp-NLS (pUMa4199)	*upp3*	AB33*upp3*Δ
**AB33*upp3*Δ::P_{otef}-mKate2-HA-L2-F2A-eGFP-NLS**	3144	*mKate2-L2-F2A-Gfp-NLS*	p*upp3*Δ::P_{otef}-mKate2-HA-L2-F2A-Gfp-NLS (pUMa4198)	*upp3*	AB33*upp3*Δ
**AB33*upp3*Δ::P_{otef}-mKate2-HA-L2-Po2A-eGFP-NLS**	3145	*mKate2-L2-Po2A-Gfp-NLS*	p*upp3*Δ::P_{otef}-mKate2-HA-L2-Po2A-Gfp-NLS (pUMa4204)	*upp3*	AB33*upp3*Δ
**AB33*rrm4*Δ/Rrm4-Gfp-L2-P2A-Firefly-HA**	3212	*rrm4-Gfp-L2-P2A-firefly-HA*	p*ip*^{R}:P_{crg}-*rrm4*-Gfp-L2-P2A-*firefly*-HA (pUMa4565)	*ip^S*	AB33*rrm4*Δ

Appendix Table S3: Plasmids generated in this study; pUMa, internal reference number

Plasmid	pUMa	Resistance cassette	Short description
**P_{rrm4}-*rrm4*-mKate2**	2985	NatR (SfiI-insert of pMF5-1n)	Vector for the expression of Rrm4 C-terminally fused to mKate2. The mKate2 cassette contains the Tnos terminator and the Nat resistance. The entire coding sequence for the fusion protein is flanked by an 830 bp upstream region and a 1.9 kb downstream region for homologous recombination. The plasmid is a derivate of pRrm4G-NatR (Becht et al., 2006), with Gfp was exchanged to mKate2.
**p*upp3*Δ::P_{otef}-mKate2-HA-F2A-Gfp-NLS**	3407	NatR	Vector contains a fusion of mKate2-HA and Gfp-NLS. mKate2 with F2A in reverse overhang was amplified with ofw: ATGGTGTCGGAGCTCATC and orev: TTTGAGGAGATCGAAGTTGAGCAGCTGTTTCACGGGG GCGTAGTCGGGCACGTCGTAAGGGTAGAGCGGACCCTGCATATGGC GGTGACCG. Gfp with F2A in forward overhang was amplified with ofw: TGAAACAGCTGCTCAACTTCGATCTCCTCAAACTGGCCGG CGACGTGGAATCAAATCCTGGACCTATGGTGAGCAAGGGC and orev: CTTGTACAGCTCGTCCATG. In between a F2A peptide ensures expression of two proteins from one open reading frame Expression is driven by the constitutive P_{otef} and terminated by T_{nos}. Flanking regions of 800 bp upstream and 700 bp downstream of the entire construct ensure homologous recombination at *upp3* locus. Plasmid was generated using AQUA cloning.
**p*upp3*Δ::P_{otef}-mKate2-HA-F2AΔP-Gfp-NLS**	3435	NatR	Same as pUMa3407. mKate2 with F2A in reverse overhang was amplified with ofw: ATGGTGTCGGAGCTCATC and orev:TTTGAGGAGATCG AAGTTGAGCAGCTGTTTCACGGGGGCGTAGTCGGGCACGTCGTAAG GGTAGAGCGGACCCTGCATATGGCGGTGACCG. Gfp with F2A in forward overhang was amplified with ofw:TGAAACAGCTGCTCA ACTTCGATCTCCTCAAACTGGCCGGCGACGTGGAATCAAATCCTGGA ATGGTGAGCAAGGGC and orev: CTTGTACAGCTCGTCCATG. In between a F2A is inserted in which the last proline is deleted. Plasmid was generated using AQUA cloning.
**pip^R::P_{oma}-*emt*1-HA-L2-P2A-Gfp-*mac*1-L2-P2A-*mac*2-3xmyc**	4131	CbxR (integration of *ip*^{R} gene into *ip*^{S} locus; ip: Iron-sulfur subunit of the suc-cinate dehydrogenase)	Vector for the expression of Emt1, Mac1 and Mac2 with respective tags, linked by the L2 linker and P2A in a single open reading frame. Emt1-HA with L2-P2A in reverse overhang was amplified with ofw:TACCTTACT CTATCAGGATCCCCGCATGAAGGTTGCCC TCCTTGC and orev: GCTCCTCGCCCTTGCTCACCATGGGACCGGGGTTCTCCTCGACGTCA CCG. *gfp-mac1* with P2A in forward overhang and L2 in reverse overhang was amplified with ofw:CGGTGACGTCGAGGAGACC CCGGTCCCATGGTGAGCAAGGGCGAGGAGC and orev:GCGGG AAGCCGTGGCTAAGCTTAAACACAGGAGCGGTCGCATCGACCG. Mac2 with L2-P2A in forward overhang and 3xmyc and Tnos in reverse overhang was amplified with ofw:ATGCGACCGCTCCTGTG TTTAAG CTTAGCCACGGCTTCCCGC and orev: GCAAAAGCGAAACAGCGG CGCGACCCTAGAGGTCCTCTTCCGAGATGAGCT TCTGCT CGGA CCACCGGCCGAGAGGTCCTCTTCCGAGATGAGCTTCTGCTCCGAGCC GGCACCGGCGAGGTCCTCTTCCGAGATGAGCTTCTGCTCCTCGGGA GCGTCAACGGGGACTG. The P2A peptide ensures translation of three proteins from a single open reading frame. Expression is driven by the synthetic constitutive promoter P_{oma} and terminated by T_{nos}. The expression cassette contains the ip*R* gene, which is cleaved to ensure homologous recombination prior to transformation.

**p*upp3*Δ::P$_{otef}$-mKate2-HA-L1-F2A-Gfp-NLS**	4261	NatR	Vector contains a fusion of mKate2-HA and Gfp-NLS. mKate2 was amplified with ofw:GGTCTCGCCTGCCCTGCAGGCTAGAACT AGTG and orev:GGTCTCCAGGCCGGCCGGCGTAGTCGGGCA CGTCGTAAGG. Gfp was amplified with ofw:GGTCTCCGCCTGC GAGAGAGAGAGCTCAGCCCATGGTGAGCAAGGGCGAGG and orev: GGTCTCGCTGCGGCGCGCCGGCCGCTAGATC. In between a F2A peptide N-terminally fused to an GSG linker (L1) ensures expression of two proteins from one open reading frame. GSG-F2A was amplified with ofw:GGCCGGCCTGGCTCGGGCCCCGTGAAACAGCTGCT CAAC and orev:GGCCGGCCTCACGGCTTCCCGCCGGCGGTGG CGGCGCAGGATGATGGCACGCTGGTCGCCATGACCGTCATGGCCTTC . Expression is driven by the constitutive P$_{otef}$ and terminated by T$_{nos}$. Flanking regions of 800 bp upstream and 700 bp downstream of the entire construct ensure homologous recombination at *upp3* locus.
**p*upp3*Δ::P$_{otef}$-mKate2-HA-L2-F2AΔP-Gfp-NLS**	4197	NatR	Same as pUMa3261. Within F2A the last proline is deleted and it is N-terminally fused to KLSHGFPPAVAAQDDGTLV (L2). F2AΔP was amplified with ofw:GGCCGGCCTCACGGCTTCCCGCCGGCGGTGG CGGCGCAGGATGATGGCACGCTGGTCCCCGTGAAACAGCTGCTCAA C and orev: GCTGAGCTCCAGGATTTGATTCCACG.
**p*upp3*Δ::P$_{otef}$-mKate2-HA-L2-T2A-Gfp-NLS**	4202	NatR	Same as pUMa4197. T2A was amplified with ofw:GGCCGGCCTCACGG CTTCCCGCCGGCGGTGGCGGCGCAGGATGATGGCACGCTGGTCCGT GGTCCTCGTCCCCAGAAC and orev:GCTGAGCGGGACCGGGGT TCTCCTCGAC.
**p*upp3*Δ::P$_{otef}$-mKate2-HA-L1-F2AΔP-Gfp-NLS**	4273	NatR	Same as pUMa4261. F2ADP was amplified with ofw:GGCCGGCCTGGC TCGGGCCGCCACAAGTTCCCCACCAAC and orev:GCTGAGCGGG ACCGGGGTTCGACTCGAC.
**p*upp3*Δ::P$_{otef}$-mKate2-HA-L2-P2A-Gfp-NLS**	4199	NatR	Same as pUMa4197. P2A was amplified with ofw:GGCCGGCCTCACGG CTTCCCGCCGGCGGTGGCGGCGCAGGATGATGGCACGCTGGTCCCC GTGAAACAGCTGCTCAAC and orev:GCTGAGCAGGTCCAGGA TTTGATTCC.
**p*upp3*Δ::P$_{otef}$-mKate2-HA-L2-F2A-Gfp-NLS**	4198	NatR	Same as pUMa4197. F2A N-terminally fused to L2 was codon optimized ordered from IDT (Integrated DNA Technologies Inc. Coralville).
**p*upp3*Δ::P$_{otef}$-mKate2-HA-L2-Po2A-Gfp-NLS**	4204	NatR	Same as pUMa4197. Po2A N-terminally fused to L2 was codon optimized ordered from IDT (Integrated DNA Technologies Inc. Coralville).
**p*ip*^{R}:P$_{crg}$-*rrm4*-Gfp-L2-P2A-*firefly*-HA**	4565	CbxR	Vector for the co-expression of Rrm4 C-terminally fused to Gfp and Firefly luciferase fused to HA tag separated during translation with L2-P2A. L2-P2A was amplified with ofw:TGTACAAACACGGCTT CCCGCCGGCGGTG and orev:CAATTGGGGACCGGGGTTCTCCT CGAC. Firefly was amplified with ofw:CGGGATCCCCCGGGCTG CAGGAATTCGATCCCCAATTGATGGAGGACGCCAAGAA and orev:GCCGGGCGGCCGGCGCGCCGGCCGCTAGATCTTTAGGCGTAGT CGGGCACGTCGTAAGGGTAGAGCGGACCCTGGACGGCGATCTTGCC.
